# Supplementary material for: A network dynamical simulation model for the study of antibiotic resistance in microbial communities
Source: Gut Microbes. 2026 Sep 19;18(1):2734703. doi: 10.1080/19490976.2026.2734703 (PMC13596919; doi:10.1080/19490976.2026.2734703)
Supplement: Supplementary file 1 [file KGMI_A_2734703_SM9053.zip › supplementary_file_1/Figures/ba_delta_gc_size_n300.pdf]

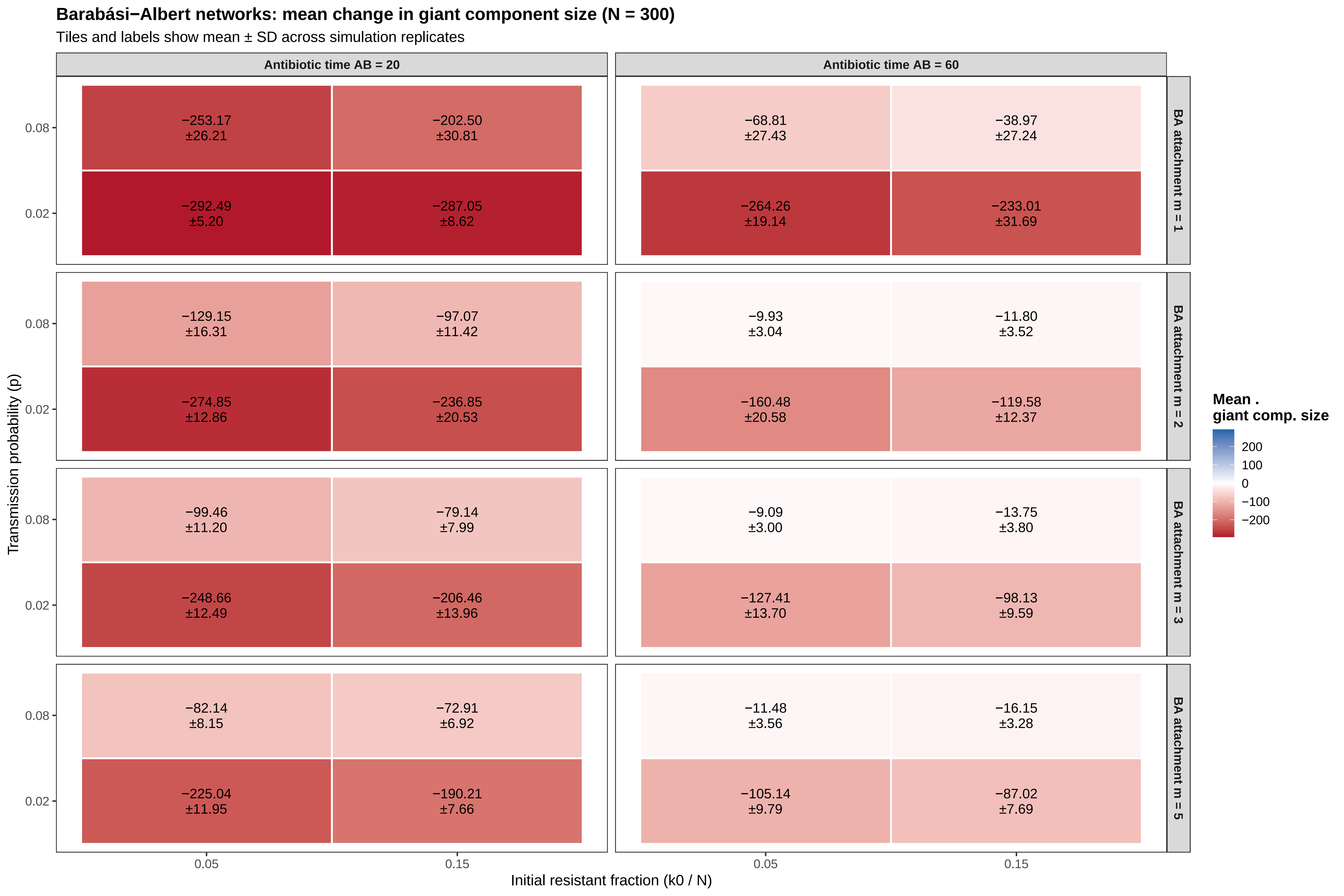

Barabási−Albert networks: mean change in giant component size (N = 300)
Tiles and labels show mean ± SD across simulation replicates
Antibiotic time AB = 20
Antibiotic time AB = 60
BA attachment m = 1
BA attachment m = 2
BA attachment m = 3
BA attachment m = 5
−253.17 ±26.21
−202.50 ±30.81
−292.49 ±5.20
−287.05 ±8.62
−68.81 ±27.43
−38.97 ±27.24
−264.26 ±19.14
−233.01 ±31.69
−129.15 ±16.31
−97.07 ±11.42
−274.85 ±12.86
−236.85 ±20.53
−9.93 ±3.04
−11.80 ±3.52
−160.48 ±20.58
−119.58 ±12.37
−99.46 ±11.20
−79.14 ±7.99
−248.66 ±12.49
−206.46 ±13.96
−9.09 ±3.00
−13.75 ±3.80
−127.41 ±13.70
−98.13 ±9.59
−82.14 ±8.15
−72.91 ±6.92
−225.04 ±11.95
−190.21 ±7.66
−11.48 ±3.56
−16.15 ±3.28
−105.14 ±9.79
−87.02 ±7.69
Transmission probability (p)
0.08
0.02
Initial resistant fraction (k0 / N)
0.05
0.15
Mean .
giant comp. size
200
100
0
−100
−200
